# Reversible Ocular Toxicity of Oxaliplatin: A Case Report

**DOI:** 10.7759/cureus.4582

**Published:** 2019-05-01

**Authors:** Arish Noor, Aakash Desai, Meghana Singh

**Affiliations:** 1 Internal Medicine, University of Connecitcut, Hartford, USA; 2 Internal Medicine, University of Connecticut, Farmington, USA; 3 Internal Medicine, University of Connecticut, Hartford, USA

**Keywords:** ocular toxicity, oxaliplatin

## Abstract

Oxaliplatin, a platinum-based chemotherapy agent, is commonly used in the treatment of various malignancies. Common adverse effects involve neurological, hematological, gastrointestinal system, and hypersensitivity, and rarely ocular changes have also been reported. We describe the case of a 71-year-old male, who developed reversible ocular toxicity after receiving oxaliplatin for treatment for pancreatic cancer.

## Introduction

Oxaliplatin, a platinum-based chemotherapy agent, is increasingly being utilized for the treatment of various solid and hematological malignancies. In combination with fluorouracil, irinotecan, and leucovorin, i.e. FOLFIRINOX, it has shown significant mortality benefit in pancreatic adenocarcinoma [1]. It has also become an integral component of colon cancer treatment [2]. Herein, we report a case of oxaliplatin-induced reversible ocular toxicity in a patient undergoing FOLFIRINOX therapy.

## Case presentation

**Figure 1:**
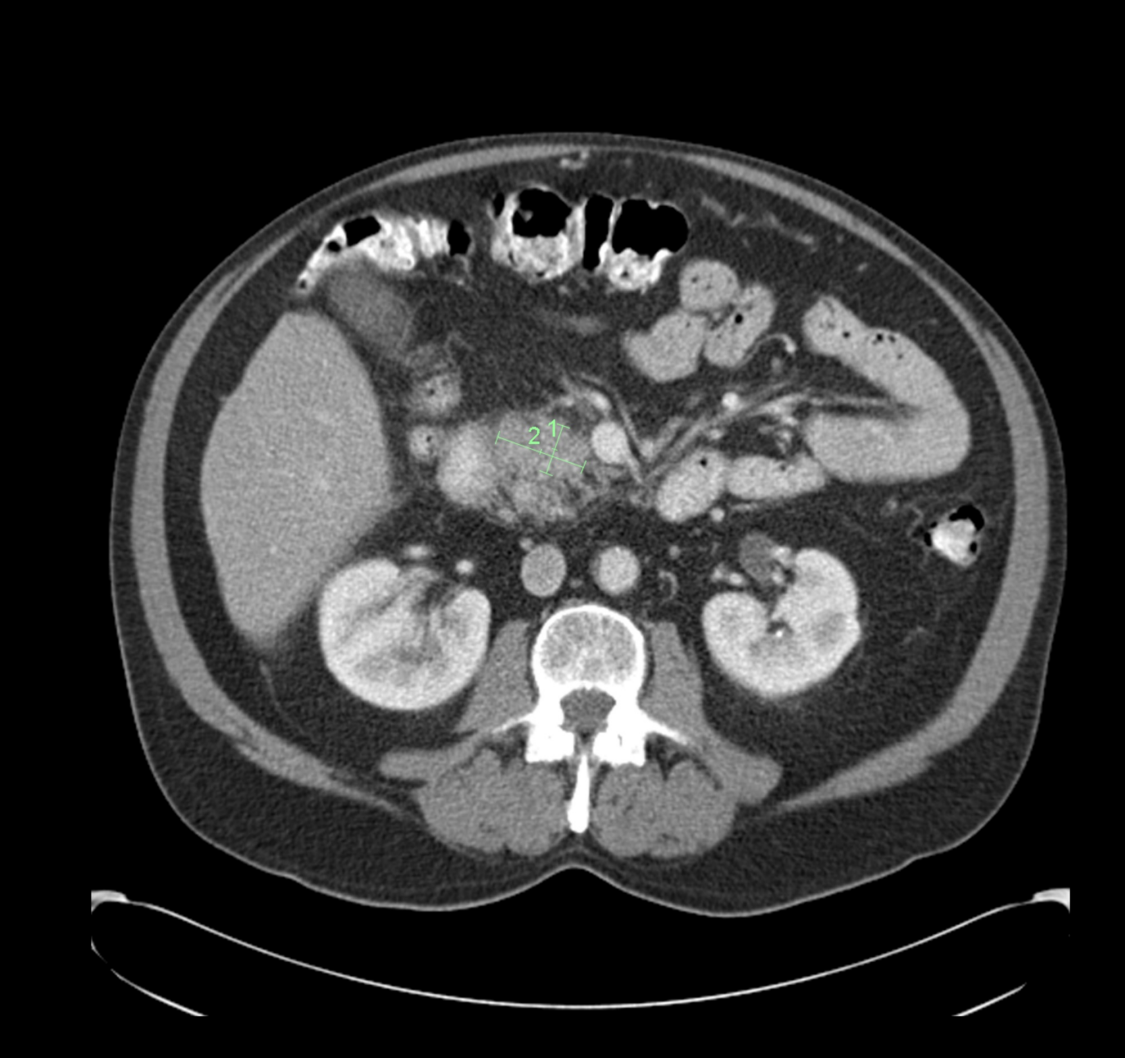
CT abdomen pelvis showing 2.0 x 1.0-cm ill-defined solid mass in the head of the pancreas CT, computed tomography

A 71-year-old man presented to the emergency department with nephrolithiasis and was noted to have abnormal liver function tests. An abdominal CT scan (Figure 1) showed a calculus in the right ureteropelvic junction and an ill-defined mass in the head of the pancreas compressing the common bile duct.

The patient then underwent an endoscopic retrograde cholangiopancreatography (ERCP) with stent placement, along with endoscopic ultrasound (EUS)-guided fine needle aspiration (FNA) of the pancreatic head. The biopsy demonstrated a pancreatic adenocarcinoma and with stage IB (T2N0M0).

The patient was taken for a Whipple surgery; however, the procedure was aborted after the discovery of liver cirrhosis on laparoscopic diagnostic liver biopsy. The patient's case was discussed in the hepatobiliary tumor board and the consensus was to proceed with systemic chemotherapy followed by chemoradiation. Induction chemotherapy using the FOLFIRINOX regimen was then initiated as part of the treatment plan. 

After starting chemotherapy with FOLFIRINOX, the patient presented with the onset of visual changes, occurring immediately following the infusion of oxaliplatin during the first cycle and prior to the complete administration of the other components of FOLFIRINOX with the second cycle. He reported a complete loss of vision in the right eye followed by tunnel vision that fully resolved within two days after the first treatment and five days after the second treatment. The patient did not report any visual changes in the left eye. A thorough ophthalmologic examination was performed, and no obvious retinal or optic nerve damage was noted. However, due to these concerning ocular manifestations, the decision was made to stop treatment with FOLFIRINOX and to switch to gemcitabine plus nab-paclitaxel. Subsequently, the patient recovered and no further visual abnormalities were reported. 

## Discussion

Oxaliplatin, a platinum-based agent, has become an integral part of treatment for various malignancies, including pancreatic and colon cancer [1-2]. Similar to other chemotherapy agents, oxaliplatin has also been associated with several adverse effects; the majority of which are related to neurotoxicity. Neurotoxicity can be acute, occurring immediately following infusion, and presents as transient paresthesia, muscular spasm, often aggravated by cold [3-4].

On the other hand, chronic neurotoxicity can manifest as sensory and distal dysesthesias and paresthesia, affecting sensorimotor coordination leading to ataxia. These effects are often cumulative, increasing over subsequent doses [3,5] 

Hematological effects, consisting of neutropenia, thrombocytopenia, and anemia have also been reported. The gastrointestinal system can be affected as well, presenting as nausea, vomiting, diarrhea, and sinusoidal injury leading to hepatotoxicity [6]. Hypersensitivity reactions, ranging in severity from mild to anaphylaxis can also occur [7-8]. There are only a few published case reports, describing ocular toxicity in the setting of oxaliplatin. This side effect is rare, often temporary and reversible. The mechanism behind the process remains unknown, however, damage of retinal pigment epithelium and optic nerve has been proposed [7].

Oxaliplatin-associated ocular symptoms broadly include conjunctivitis, abnormal lacrimation, blurred vision, visual loss, tunnel vision and abnormalities with color perception have been reported [9]. Occasionally permanent changes such as retinal damage and loss of visual fields have also been reported. Oncology nursing society also reports cataracts, retinal opacities, optic neuritis, inflammatory conditions such as blepharitis, uveitis, keratitis, iritis, and conjunctivitis [10].

A retrospective study carried out in Japan examined oxaliplatin-induced ocular toxicity in 55 people. 18.2% had visual problems. Blepharoptosis occurred in 9.1%, visual field defects in 3.6%, decreased visual acuity in 3.6%, ocular pain in 1.8%, congestion in 1.8%, excessive lacrimation 1.8% and blurred vision in 1.8%. These symptoms were mild and occurred early during the administration of chemotherapy; mostly with the first or second sessions [11]

A published case series provided further supporting evidence. The authors reported a case of oxaliplatin-induced ocular toxicity, that started as peripheral vision loss and progressed to the central vision. Associated funduscopic findings consisted of bilateral papilledema with areas of hemorrhage and optic neuritis affecting both eyes. Two other cases of tunnel vision and another case of complete bilateral visual loss were also described [12].

Another study with 67 patients found to have temporary blurred vision (13%), visual field cuts (13%), ptosis (13%) and eye pain (20%) among patients with oxaliplatin-induced visual changes [4]. One of the cases reported the occurrence of blurred vision, altered color sensation along with neurological symptoms, in a 52-year-old female receiving FOLFOX -4 for colon cancer. These changes were temporary and resolved within three weeks of agent discontinuation [13].

## Conclusions

Our case highlights the rare side effect of oxaliplatin and emphasizes the importance of detecting ocular symptoms early during the course of chemotherapy regimen, especially in those receiving oxaliplatin. Any abnormality in the visual function needs to be immediately addressed with the patient being referred to an ophthalmologist for a detailed examination.
